# Enhancing group outcomes: the role of individual preparation in collaborative learning

**DOI:** 10.1186/s12909-025-06925-1

**Published:** 2025-04-12

**Authors:** Min Hae Song, Jaeseo Lim, Seunghee Lee, Jungjoon Ihm, Jooyong Park

**Affiliations:** 1 Department of Psychology, Seoul National University, Seoul, Korea; 2 Department of Counseling Psychology, Jeonju University, Jeonju, Korea; 3 Department of Medical Education, College of Medicine, Seoul National University, Seoul, Korea; 4 Department of Dental Education, Dental Research Institute, School of Dentistry, Seoul National University, Seoul, Korea

**Keywords:** Collaborative learning, Individual preparation, Learning effect, Instructional approach

## Abstract

**Background:**

Individual preparation can enhance the effects of collaborative learning through effective information processing. However, the limitations of prior studies in distinguishing the unique effects of individual preparation from individual learning, as well as their focus on short-term effects within the humanities and social sciences, have hindered its application into education for health professionals. Therefore, the current study examined the distinct effects of individual preparation for collaborative learning in short- and long-term contexts by conducting two experiments.

**Methods:**

Experiment 1, conducted with 88 college students, compared the immediate and long-term test results of participants who prepared individually before collaborating, those who engaged in collaborative learning, and those who engaged in individual learning. Experiment 2, conducted with 71 medical and dental students, examined the learning effect of individual preparation for collaborative learning using medical education materials, in comparison to collaborative learning and individual learning.

**Results:**

In Experiment 1, the results demonstrated that the participants who prepared individually before collaboration performed better than those who engaged solely in collaborative or individual learning. The results of Experiment 2 indicated that medical and dental students who prepared individually and then collaborated performed better than those who engaged solely in collaborative or individual learning in short- and long-term settings.

**Conclusions:**

These findings suggest that individual preparation for collaborative learning can be an effective instructional approach across educational settings, which enhances the academic performance in short- and long-term perspectives especially in education for health professionals.

**Supplementary Information:**

The online version contains supplementary material available at 10.1186/s12909-025-06925-1.

## Background

In this rapidly changing era, education should prioritize enhancing students' ability to transfer and apply knowledge, rather than focusing solely on shallow knowledge acquisition. To align with these educational demands, universities are increasingly adopting various instructional approaches that promote the application and transfer of knowledge [1–3]. Among these approaches, collaborative learning, where students work together on a common goal or task, has gained significant attention for its potential to enhance critical thinking, problem-solving, and the ability to apply knowledge [3–6].

Despite its promise, collaborative learning does not always yield superior learning outcomes compared to individual learning. Previous studies have shown that collaborative learning can sometimes result in lower learning gains due to its increased complexity and higher cognitive demands, which may hinder retrieval and inference processes during collaboration [7–10]. To address this issue and then provide empirical evidence for expanding the implementation of collaborative learning in education, we propose that students should prepare individually prior to engaging in collaborative learning by assigning tasks or questions [11–16]. While the effect of individual preparation has been explored in general educational settings, its application and efficacy within medical education remain underexplored. Given the high stakes and unique demands of health professional training, understanding the role of individual preparation in optimizing collaborative learning is particularly relevant. This study aims to address this gap by investigating the impact of individual preparation on collaborative learning outcomes, with a focus on both immediate and long-term knowledge retention in the context of health professional education.

The benefits of individual preparation for collaborative learning can be explained using information processing theory. Collaborative learning involves interactive activities that require simultaneous retrieval, inferencing, and referencing of learning materials [17]. Since students should work simultaneously during these activities, collaborative learning imposes a higher cognitive load compared to individual learning [9, 11, 12, 17]. Additionally, disruptions that occur during interactions, such as production blocking and retrieval strategy interference, further increase the cognitive load, potentially hindering deeper engagement in collaborative learning [11, 12, 17].

However, individual preparation can effectively reduce these transactional costs and cognitive loads by allowing students to deal with the learning material beforehand. This preparation enables students to engage more effectively in interactive activities with a reduced cognitive load and better information processing than if they were to begin collaborating without individual preparation [9, 15]. Consequently, researchers have recognized individual preparation as an effective method for enhancing the outcomes of collaborative learning [16].

Several gaps remain in the existing research on individual preparation for collaborative learning, which should be addressed before its broad adoption in educational settings. Previous research primarily focused on confirming its effectiveness compared with that of immediate collaboration alone, which makes identifying its unique effects challenging [1, 15, 18, 19]. These results can make opting for individual preparation for collaborative learning instead of individual learning in class difficult for instructors.

Korde and Paulus examined the distinct effects of individual preparation for collaboration [20]. They found that significantly more ideas were generated when participants collaborated after individual preparation than they collaborated immediately. However, no significant difference was found between the number of ideas generated individually and those generated through collaboration after individual preparation. These findings suggested that the learning effect of individual preparation for collaborative learning may not significantly differ from learning individually, potentially reducing the utilization of individual preparation for collaborative learning. Therefore, exploring the unique effects of individual preparation distinguished from collaborative and individual learning is needed.

Moreover, we anticipate that individual preparation for collaborative learning could offer significant benefits in health professional education. The evolving demands of today’s healthcare environment require health professionals not only to possess foundational knowledge but also to develop critical thinking and problem-solving skills essential for making optimal decisions in patient care. Given these requirements, collaborative learning enhanced by individual preparation may provide a valuable approach. However, previous studies have primarily focused on immediate learning effects using materials from the humanities and social sciences [11, 12, 15, 16], making it difficult to determine whether this approach is suitable for health professional education. Therefore, we explored whether individual preparation for collaborative learning is effective in health professional education over both the short and long term.

To address these gaps, this study aims to:

1. Substantiate the distinctive impact of individual preparation for collaborative learning by comparing it with individual and collaborative learning.
2. Examine the short- and long-term learning effects of individual preparation using learning material from health professional education.

### Overview of the experimental design

To investigate the effectiveness of individual preparation for collaborative learning in medical education, we designed two experiments. Experiment 1 serves as a background study to replicate and extend prior findings on individual preparation using structured tasks. The findings from Experiment 1 informed the design of Experiment 2, which involved more complex, ill-structured tasks to examine the effects of individual preparation in problem-based learning scenarios. By increasing the task complexity in Experiment 2, we sought to simulate the demands of real-world medical education and explore how individual preparation supports learning in such contexts. Together, these experiments aim to provide a comprehensive understanding of how individual preparation impacts short- and long-term learning outcomes in health professional education.

### Research questions

This research seeks to answer the following questions:

1. What are the unique effects of individual preparation on collaborative learning compared to individual and collaborative learning alone?
2. Does individual preparation enhance both short- and long-term learning outcomes in health professional education?

By systematically addressing these questions, we aim to provide empirical evidence to support the broader implementation of individual preparation in collaborative learning, particularly in the context of health professional education.

## Experiment 1

We examined the unique effect of individual preparation followed by collaborative learning compared with collaborative and individual learning. Therefore, we divided participants under three conditions based on learning activities: individual preparation for collaborative learning (IP), collaborative learning alone (C), and individual learning (I). The dependent measure was the score on the final test, consisting of comprehension and transfer questions.

### Methods

#### Participants

Before the experiment, we conducted a pilot test with 24 participants, which yielded an effect size of (*η*_*p*_^*2*^ = 0.13). Based on this, we performed a G*Power priori power analysis effect size (*η*_*p*_^*2*^ = 0.13) with an alpha of 0.05 and a power of 0.90. The G*Power analysis recommended that we use 87 participants (29 participants per condition). Therefore, we chose to have at least 29 participants per condition. We recruited 88 undergraduate students at a university in Seoul, Korea, to the experiment in exchange for course credit. The participants (men: 50, women: 38) were of Asian descent, specifically Korean. This specificity was due to the utilization of learning materials written in the Korean language. They were recruited through the online recruiting system of the Department of Psychology. The participants provided informed consent, which was approved by the Institutional Review Board (IRB No.1804/003–005) of the university. Five participants with prior knowledge were excluded from analysis. Therefore, data from 83 participants (mean age = 20.30 years; *SD* = 2.05) were analyzed.

Each participant was randomly assigned to under one of the three conditions: IP (*n* = 29), C (*n* = 30), and I (*n* = 24). We randomly assigned groups of three or four participants to the IP and C conditions. On the contrary, participants under the I condition worked on problems individually.

#### Learning material

Participants studied the subject “criminal procedure code and the accusation and charge” using seven pages of written material utilized in prior research [21]. We expected that this topic will minimize the possibility of the influence of prior knowledge on the final test, because the university where participants attend does not offer law courses for undergraduate students. Nevertheless, there could have been participants who were familiar with the selected topic, which may influence the scores [22, 23]. A background knowledge survey using a 7-point Likert scale, ranging from 1 (having no knowledge) to 7 (having expert knowledge), was conducted prior to the experiment. Five participants who reported a point higher than 4, indicating that they had previously studied the topic, were excluded from the final analysis.

#### Learning activities

The participants under the three conditions drew a concept map that included a summarization of the learning material and three or more questions about the content. The procedure for each condition was as follows: under the IP condition, participants initially worked on a concept map individually for the first 9 min. Three to four students formed a group then collaborated to draw a concept map together for 11 min based on the concept maps of each member. Under the C condition, three to four participants collaborated for 20 min to create a concept map from the beginning. Lastly, under the I condition, participants independently drew a concept map for 20 min.

#### Test questions

We utilized a total of 10 questions, consisting of six comprehension questions and four transfer questions from Lim and Park, which are directly related to the learning material [21]. The total score was 40 points with 22 points allocated for six comprehension questions and 18 points for four transfer questions. Appendix 1 provides the sample questions for each type.

Below is an example of the predetermined partial points and scoring manual in comprehension questions. For instance, a comprehension question from Appendix 1 asked participants to explain who is entitled to file a complaint. This question was worth 3 points, with partial points assigned as follows: (1 point) "no one to make the accusation," (1 point) "prosecutors shall designate the person with the right to file a complaint," and (1 point) "within 10 days upon request by stakeholders." Raters scored predetermined partial points whenever the keywords from each response were included.

In contrast, answering the four transfer questions required participants to engage in deeper thinking about the learning material. And they were also open-ended and the scoring for these questions considered whether the responses included essential components aligned with the learning objectives, with specific criteria predetermined as part of the scoring process.

Below is an example of the predetermined partial points and scoring manual in transfer questions. For instance, a transfer question from Appendix 1 was worth 5 points, with partial points assigned as follows: (1 point) whether the victim (V) was underaged was considered, (1 point) whether the participants understood that sexual crimes are not offenses subject to complaint, (1 point) whether participants recognized that accusation by the victim is not required for prosecution, (1 point) whether participants recognized that accusation by the legal representative is not required for prosecution, and (1 point) whether the conclusion "the court of appeals will reject D’s claim" was derived. Raters referred to this manual to determine whether each element was present in the responses and assigned scores accordingly.

To ensure scoring consistency and reliability, raters were also trained before scoring the participants’ responses. During training, raters reviewed manuals similar to those described above and practiced scoring using five sample responses. They discussed discrepancies to ensure a shared understanding of the criteria and alignment in their evaluations.

#### Procedure

**Fig. 1:**
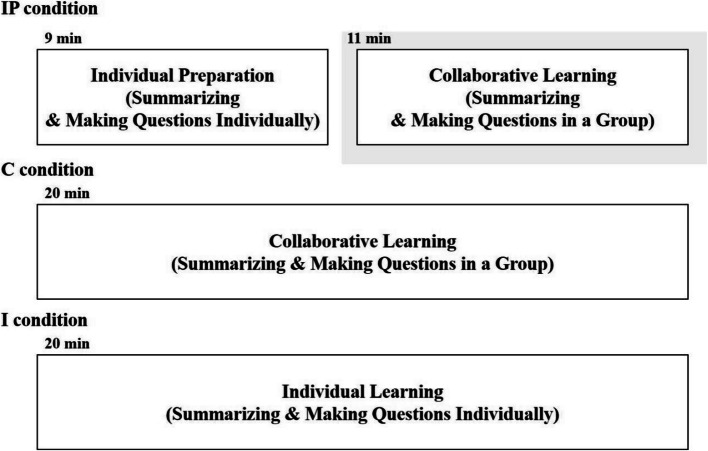
Detailed procedures of learning activities for each condition in Experiment 1

Experiment 1 was conducted in a laboratory. Participants were firstly required to take the background knowledge survey. Afterward, they individually studied the learning material for 10 min. They then summarized the content and created at least three questions related to the subject on a concept map for 20 min according to the assigned condition. The experimenter provided no guidance on the collaboration process in order to minimize potential influence. Participants were allowed to review the learning material while creating concept maps. Finally, they took the final test for 15 min without access to the learning material. Figure 1 depicts the procedure of Experiment 1.

#### Analysis

We conducted an ANOVA and Tukey’s HSD to determine the learning effect of each learning condition. Two raters graded the scores used in the analysis to ensure reliability in grading. The scores used for analysis were initially rated by a single rater, the first author, who specializes in educational psychology. To ensure scoring reliability, a second rater—a practicing lawyer in the Republic of Korea—independently evaluated over 50% of the total responses (42 responses). We then measured the intraclass correlation coefficient (ICC) of the scores graded by both raters. The agreement between the raters, as measured by the ICC, was 0.93. This indicates that the grading of the first rater could be sufficiently trusted. Statistically significant differences were denoted by *p*-values < 0.05. The effect sizes of ANOVA were confirmed using partial eta square (*η*_*p*_^2^).

#### Results and discussion

Table 1 displays the mean and standard deviation of the scores. ANOVA displayed a statistically significant difference among the three conditions (*F*(2, 80) = 8.98, *p* < 0.001, *η*_*p*_^2^ = 0.18). Tukey’s HSD shows that there were significant differences between the IP condition and I conditions (*p* < 0.001) and IP and C conditions (*p* = 0.033), but no difference between the C and I conditions (*p* = 0.180). Therefore, participants in the IP condition outperformed those in the C and I conditions.

**Table 1:** Mean and standard deviation of test score by learning condition

	IP	C	I
Total score	27.59 (*3.79*)	24.37 (*5.87*)	22.00 (*4.50*)
Comprehension score	17.66 (*2.38*)	16.23 (*3.84*)	14.04 (*4.14*)
Transfer score	9.93 (*3.04*)	8.30 (*3.21*)	7.96 (*2.80*)

Mean values and standard deviations (*SD* values are enclosed in parentheses)

To determine the cause of the difference in the total score, we conducted ANOVA on comprehension and transfer scores. The results indicated that significant differences exist among the three conditions (comprehension: *F*(2, 80) = 7.03, *p* = 0.001, *η*_*p*_^2^ = 0.15; transfer: *F*(2, 80) = 3.35, *p* = 0.040, *η*_*p*_^2^ = 0.08). Therefore, we confirm that individual preparation for collaborative learning leads to better learning outcome to either collaborative or individual learning.

Since our results, like those of previous studies, indicate that individual preparation for collaborative learning is an effective learning method, this approach could potentially be applied to education for health professionals. However, as Experiment 1 used only learning material from law subject with students from various disciplines, it is necessary to conduct an experiment specifically targeting medical and dental students using educational material relevant to medical education. Therefore, Experiment 2 aims to replicate the results using learning material from education for health professional and task with medical and dental students.

## Experiment 2

We investigated the immediate and long-term learning effects of individual preparation within the medical and dental students. We used problem-solving tasks instead of concept mapping which was used in Experiment 1 and forensic learning material from education for health professionals. Participants were divided into three conditions (i.e., IP, C, and I) based on their learning activities, same with Experiment 1. The dependent measures were the scores on immediate and long-term tests, which included comprehension and transfer questions.

### Methods

#### Participants

Based on the results of Experiment 1, we conducted a G*Power priori power analysis, with an effect size (*η*_*p*_^*2*^ = 0.16) calculated as the average effect size from the total score and comprehension score with an alpha of 0.05 and a power of 0.90. The G*Power analysis recommended that we use 72 participants (24 participants per condition). Therefore, we chose to have 24 participants per condition. We recruited 71 undergraduate students (medicine: 25, dentistry: 46) at a university in Seoul, Korea. All participants (men: 49, women: 22) were of Asian descent, specifically Korean. This specificity was due to the utilization of learning material written in the Korean language. The participants provided informed consented before the study, which was approved by the Institutional Review Board (IRB No.S-D20220010) of the university. The mean age of the participants was 19.10 years (*SD* = 0.97).

Each participant was randomly assigned under one of the three conditions: IP (*n* = 21), C (*n* = 25), and I (*n* = 25). To reduce potential bias due to familiarity, we randomly assigned groups of three or four participants under the IP and C conditions after obtaining the student lists from class instructors and anonymizing them. Participants under the I condition individually solved the problems.

#### Learning material

The participants were provided with five pages of written material on the subject of forensic science. It included descriptions of concepts on types of wounds, poisoning, and vital reactions. We selected this material, because we deemed that the topic would minimize the possibility that prior knowledge would influence the final test results. The reason is that the university where participants attend does not offer forensic science courses for undergraduate medical and dental students. Similar to Experiment 1, a background knowledge survey using a 7-point Likert scale was conducted prior to the experiment. All participants reported that they had not previously studied the topic.

#### Learning activities

**Fig. 2:**
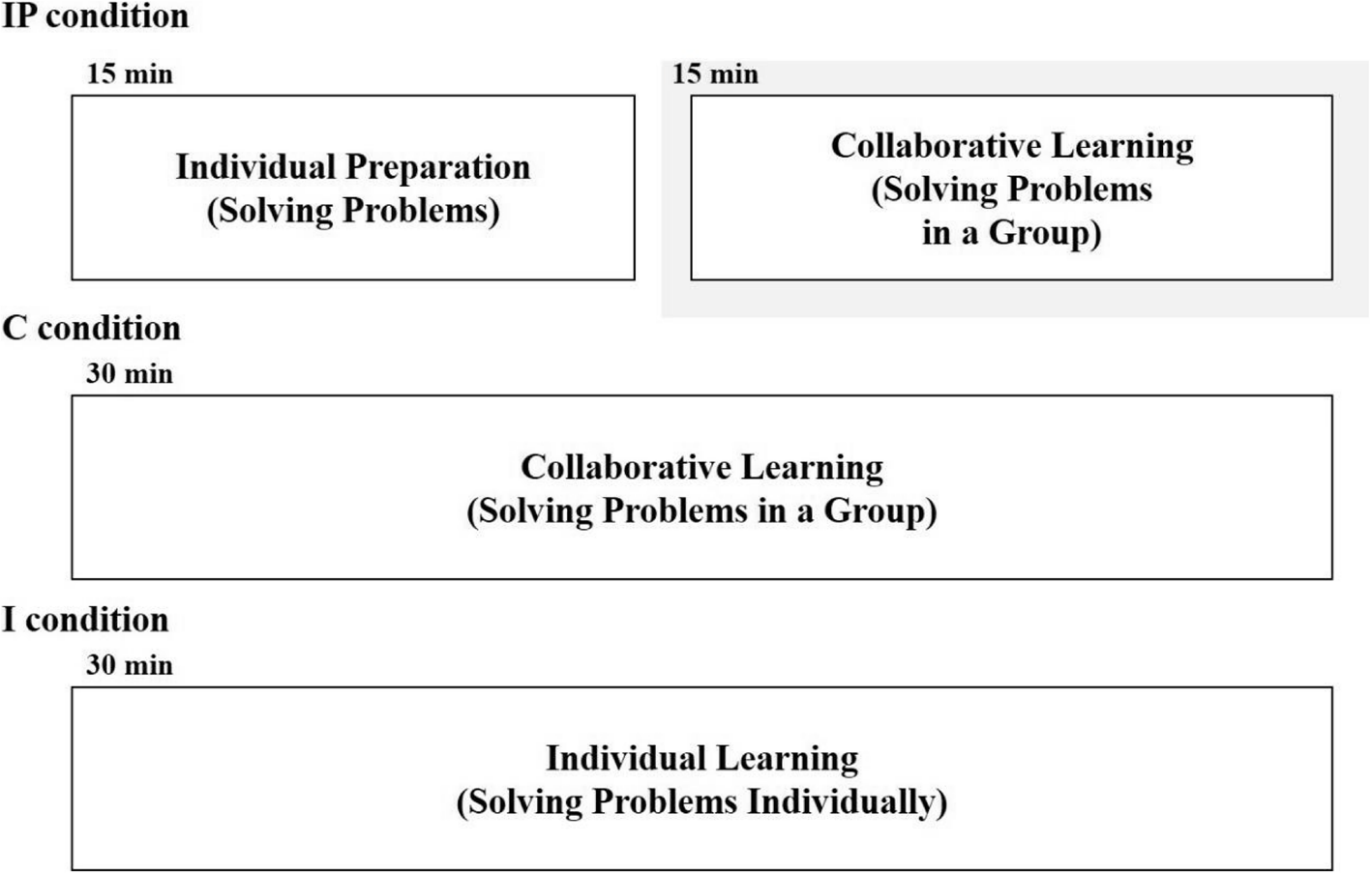
Detailed procedures of learning activities for each condition in Experiment 2

The participants solved problem, which required them to identify the types of wounds and poisons based on various factors for learning according to the assigned condition. Appendix 2 presents the sample problems. Under the IP condition, participants first solved problems individually for 15 min. They then collaborated to solve problems considering the outcomes produced by all group members during the individual preparation session. Through critical discussion and evaluation of each member’s problem-solving approaches, the group worked together to develop a single, agreed-upon solution for each problem. The groups consisted of three or four participants, and collaborative learning lasted 15 min. Under the C condition, three to four participants solved the problem together for 30 min. This condition required participants to engage in collaborative learning from the beginning to produce a single, agreed-upon solution, consistent with traditional collaborative learning practices. Finally, under the I condition, participants solved the problem individually for 30 min. Figure 2 provides the detailed procedures for each condition.

#### Test questions

The participants took two tests: the first was immediately taken after learning (immediate test), and the second was conducted one week later (long-term test). Both tests included comprehension and transfer questions and were scored using predetermined partial points based on the guidelines of the administrative examination in Korea, as the questions for both tests were derived from this examination. The author and another expert with a background in medicine evaluated the scores anonymously, excluding personal information and experimental conditions. The level of difficulty and the total scores for both tests were the same. Both included five comprehension and four transfer questions. The comprehension questions assessed understanding of the learning material, while the transfer questions required deeper thinking. Appendix 2 provides the sample questions for each type.

#### Procedure

The participants performed the experiment individually or collaboratively in the class. First, participants were required to complete the background knowledge survey. Afterward, they studied the material individually for 15 min. They were only allowed to study using the provided learning material and could not use other resources, such as the internet or textbooks. They then solved the problems for 30 min according to their assigned condition. The experimenter provided no guidance on the collaboration process to minimize potential influence. Participants were allowed to review the learning material while solving problems. Afterward, participants rated the efficiency and difficulty of the learning method using a 7-point Likert scale followed by the immediate test, which lasted for 15 min. Finally, after one week, participants took the long-term test for 15 min.

#### Analysis

We conducted an ANOVA and Tukey’s HSD to determine the learning effect of each learning condition. The scores used for analysis were initially rated by a single rater, the first author, who specializes in educational psychology. To ensure scoring reliability, a second rater— a master’s student in the medical field who received prior training —independently evaluated over 50% of the total responses (36 responses). The resulting ICC of 0.91 confirmed the reliability of the scoring.

#### Results and discussion

Table 2 displays the mean and standard deviation of the immediate test score, perceived efficiency and difficulty**.** ANOVA indicated that there were no significant differences in perceived efficiency and difficulty among the three conditions (perceived efficiency: *F*(2, 68) = 0.02, *p* = 0.985, *η*^*2*^ = 0.00; perceived difficulty: *F*(2, 68) = 1.52, *p* = 0.227, *η*^*2*^ = 0.04). Although the IP condition required more effort from the students than the other conditions, there were no differences in perceived difficulty (Table 2).

**Table 2:** Mean and standard deviation of immediate test score, perceived efficiency and difficulty by learning condition

	IP	C	I
Total score	20.48 (*4.92*)	17.68 (*4.53*)	15.92 (*4.56*)
Comprehension score	10.00 (*3.26*)	8.36 (*2.58*)	7.68 (*2.67*)
Transfer score	10.29 (*3.04*)	9.32 (*2.91*)	8.24 (*2.73*)
Perceived efficiency	4.38 (*1.02*)	4.36 (*1.19*)	4.32 (*1.44*)
Perceived difficulty	2.29 (*1.10*)	2.88 (*1.13*)	2.64 (*1.22*)

Mean values and standard deviations (*SD* values are enclosed in parentheses)

ANOVA indicated a statistically significant difference in the total scores for the immediate test among the three conditions (*F*(2, 68) = 5.51, *p* = 0.006, *η*_*p*_^2^ = 0.14). Tukey’s HSD shows that there was a significant difference between the IP and I conditions (*p* = 0.004), but no differences between the C and I conditions (*p* = 0.379) or between the IP and C conditions (*p* = 0.112). Although the IP condition did not significantly outperform the C condition, participants in the IP condition still did better than those in the C condition.

To pinpoint the cause of the difference in the total scores, we conducted ANOVA on the comprehension and transfer questions. We found significant differences (comprehension: *F*(2, 68) = 3.99, *p* = 0.023, *η*_*p*_^2^ = 0. 11; transfer:* F*(2, 68) = 3.60, *p* = 0.033, *η*_*p*_^2^ = 0.10). These results indicate that individual preparation for collaborative learning exhibit high learning effect in the short-term.

Table 3 displays the mean and standard deviation of the long-term test score. ANOVA showed a significant difference in the total scores for the long-term test under the three conditions (*F*(2, 57) = 5.91, *p* = 0.004, *η*_*p*_^2^ = 0.17). Similar to the result of the immediate test, Tukey’s HSD shows that there was a significant difference between the IP condition and I conditions (*p* = 0.003), but no differences between the C and I conditions (*p* = 0.229) or between the IP and C conditions (*p* = 0.123). However, participants in the IP condition scored higher than those in the other two conditions. To identify the cause of the difference, we conducted ANOVA for the comprehension and transfer scores. We found significant differences in both questions (comprehension: *F*(2, 57) = 5.20, *p* = 0.008, *η*_*p*_^2^ = 0.15; transfer: *F*(2, 57) = 3.21, *p* = 0.048, *η*_*p*_^2^ = 0.10). Taken together, these results demonstrate that individual preparation for collaborative learning enhances academic performance of medical and dental students in the short- and long-term.

**Table 3:** Mean and standard deviation of long-term test score by learning condition

	IP	C	I
Total score	16.72 (*3.77*)	13.88 (*4.57*)	11.5 (*5.32*)
Comprehension score	7.56 (*3.05*)	5.29 (*2.65*)	4.89 (*2.42*)
Transfer score	9.17 (*2.01*)	8.54 (*3.58*)	6.61 (*3.55*)

Mean values and standard deviations (*SD* values are enclosed in parentheses)

## General discussions

We investigated the learning effects of individual preparation for collaborative learning, comparing it with both collaborative and individual learning, through two controlled experiments. Furthermore, we explored the potential application of individual preparation for collaborative learning in health professional education.

In Experiment 1, participants who prepared individually before engaging in collaborative learning scored significantly higher than those who engaged solely in collaborative or individual learning, suggesting potential advantages of individual preparation for collaborative learning in the context of law, aligned with results of previous research [12, 17, 21]. In Experiment 2, conducted with medical and dental students using forensic science materials, participants who prepared individually before collaborating showed superior academic performance on both immediate and long-term tests compared to those in the individual learning group. Although these participants did not significantly outperform those in the collaborative learning condition, they consistently achieved higher scores on both tests.

The results of Experiments 1 and 2 can be attributed to the advantages of individual preparation, which facilitates the acquisition of prior knowledge and promotes deeper engagement in collaborative learning [12, 15–17]. Thus, these results suggest that collaborative learning with individual preparation may offer benefits over collaborative and individual learning alone across disciplines, particularly in health professional education. Furthermore, the study highlights the positive effects of individual preparation on both immediate and long-term tests.

This study offers several important implications for health professions education. First, by comparing individual preparation for collaborative learning with individual and collaborative learning alone in a controlled setting, we expand on previous research on individual preparation for collaborative learning in educational psychology. Collaboration with individual preparation is often viewed as a combination of individual and group performance [17]; however, prior studies that did not directly compare this approach to collaboration alone may have failed to capture its full effects. Our results provide valuable insights into the distinct benefits of individual preparation for collaborative learning over purely individual learning or collaborative learning.

Our study may appear similar to the experiments conducted by Korde and Paulus, who focused solely on the number of ideas generated during collaboration [20]. However, their findings are difficult to apply directly to educational settings, as education requires consideration of both immediate learning outcomes and subsequent achievements. By focusing on test results as subsequent achievements of collaborative learning with individual preparation, our findings broaden the scope of research on individual preparation for collaborative learning within educational psychology.

Second, we demonstrate that individual preparation enhances the learning effects of collaborative learning across materials in both law and medical education. In both experiments, participants who engaged in individual preparation for collaborative learning outperformed those involved in only collaborative or individual learning on comprehension and transfer questions. These findings align with prior research, suggesting that individual preparation activates and elaborates on prior knowledge, resulting in higher comprehension scores [11, 12, 17]. Additionally, the in-depth interactions fostered by individual preparation led to improved transfer scores.

Furthermore, participants who prepared individually for collaborative learning achieved the highest scores on long-term tests, providing preliminary evidence that the benefits in comprehension and transfer might be sustained over time. This enduring effect may be due to enhanced retrieval, as noted in studies comparing individual preparation for collaboration with immediate collaboration in memory settings [24, 25]. This finding is particularly relevant in medical education, where the ability to apply knowledge in the long term is essential.

Finally, this study provides practical implications that could be applicable across various educational fields, particularly in health professional education, with relatively minimal additional burden on instructors. While instructors often intervene actively in the collaboration process to enhance the effectiveness of collaborative learning, these approaches can be costly [17]. However, our proposed design requires only a simple allocation of time between individual preparation and collaboration, reducing the instructional burden and making it a viable option for health professional education. Therefore, we expect that our study can broaden the applicability of individual preparation for collaborative learning across disciplines, especially in health professional education, and benefit a diverse range of students.

While we have established the learning effects of individual preparation for collaborative learning and identified its potential for application in health professional education, further research is needed to explore these effects in more detail and assess their practical applications. For example, a qualitative analysis of collaborative interactions like the discussion analysis of prior studies [13, 14, 21] could clarify the differences between individual preparation and immediate collaboration. This study did not record participant interactions, which limited our ability to conduct a qualitative analysis. Future studies could address this gap to uncover the underlying mechanisms.

It is also necessary to examine the learning effects of individual preparation for collaborative learning in real educational settings with varied tasks. In this study, participants collaborated in a controlled experimental environment with unfamiliar peers and only once. Classroom settings, where students know each other and collaborate over extended periods, such as a semester, may yield different results from those suggested from a meta-review [17]. This is especially relevant in medical education, where the application of knowledge in high-stakes, practical settings (e.g., surgical simulations) is crucial. Verifying the effects of individual preparation for collaborative learning in natural classroom environments over the long term will be essential to support its broader adoption in medical education and beyond.

## Conclusion

The findings demonstrate that individual preparation for collaborative learning has positive effects in both the short and long term for all students, particularly medical and dental students. This study provides valuable insights into the distinct benefits of preparing individually before engaging in collaborative learning, compared to either collaborative or individual learning alone, which holds the potential to significantly impact health professional education. We hope that this study will encourage the broader adoption of individual preparation for collaborative learning in health professional education, thereby enriching students' learning experiences.

## Supplementary Information

Supplementary Material 1.

## Data Availability

Data are not publicly available due to restrictions from the Institutional Review Board but available from the corresponding author upon reasonable request.

## Abbreviations

IP: Individual preparation for collaborative learning condition
C: Collaborative learning alone condition
I: Individual learning condition
