# Supplementary material for: Enhancing group outcomes: the role of individual preparation in collaborative learning
Source: BMC Med Educ. 2025 Apr 12;25:524. doi: 10.1186/s12909-025-06925-1 (PMC11992731; doi:10.1186/s12909-025-06925-1)
Supplement: Supplementary file 1 — Supplementary Material 1. [file 12909_2025_6925_MOESM1_ESM.docx]

**Appendix**

**Appendix 1**

**Sample comprehension question**

1. Explain who is the person entitled with the right to file a complaint.

(Answer: Provided that there is no one to make the accusation, prosecutors shall designate the person with the right to file a complaint within 10 days upon request by stakeholders.)

**Sample transfer question**

1. The under-aged victim (V) accused person D of rape, but eventually withdrew her accusation on February 1, 2017. Afterward, V’s father (F), legal representative of V, accused D on February 10, 2017. D was charged with rape and was convicted of the crime on the first trial. However, D made an appeal claiming that F’s complaint should not take effect since V already withdrew her complaint; thus, the prosecutor’s indictment was against the provisions of the law. Will the Court of Appeals accept D’s claim?

(Answer: Sexual crimes are not an offense subject to complaint; thus an accusation by the victim or a legal representative is not required for prosecution. Thus, the Court of Appeals will reject D’s claim.)

**Appendix 2**

**Sample question**

A couple was discovered deceased in the master bedroom of their home. The wife’s face appeared congested, and numerous small haemorrhages were visible in the conjunctiva surrounding her eyelids and eyes. Her hands and chin had multiple bruises, and several 1-cm-long cuts or abrasions were evident on the front of her neck. The husband’s body was found next to her with a knife in his right hand, and his fingerprints were apparent on the handle. There were five shallow parallel cutting wounds on the right side of his neck, barely enough to pierce the skin. The husband had similar 1-cm-long cuts or abrasions on his arms and face. His mouth contained foam, and a strong odor of decaying apricot kernels was detected. The mucous membranes of his lips appeared red and corroded, and livor mortis seemed to be slightly dark pink.

1. Explain the probable cause of the death of the husband.
